# Supplementary material for: COVID-19 is associated with higher risk of venous thrombosis, but not arterial thrombosis, compared with influenza: Insights from a large US cohort
Source: PLoS One. 2022 Jan 12;17(1):e0261786. doi: 10.1371/journal.pone.0261786 (PMC8754296; doi:10.1371/journal.pone.0261786)
Supplement: S1 File — (DOCX) [file pone.0261786.s001.docx]

# SUPPLEMENT

Original document: **COVID-19 is associated with higher risk of venous thrombosis, but not arterial thrombosis, compared with influenza: Insights from a large US cohort**

Methods - Additional Details

Since the vast majority of strokes, MIs, and PEs require inpatient treatment, the identification of these endpoints were restricted to inpatient settings to maximize the accuracy of data capture. Inpatient diagnoses were identified as those which were attached to a claim which originated from an inpatient hospital (CMS place of service 21)[[1]](https://paperpile.com/c/h4aej8/2OKz). However, because a significant percentage of acute DVTs do not require inpatient hospitalization [[2]](https://paperpile.com/c/h4aej8/TzRQw), the identification of acute DVT was expanded to the emergency department setting (CMS place of service 23) as well as the inpatient setting. The identification of secondary endpoints (angina, transient ischemic attack, peripheral arterial disease, amputation, coronary angioplasty, coronary artery bypass grafting, venous thrombosis of devices, implants, or grafts) were similarly restricted to ICD codes attached to claims which originated from either an inpatient hospital or emergency room (CMS place of service 21 or 23) [[1]](https://paperpile.com/c/h4aej8/2OKz).

COVID-19 ICD diagnosis codes and LOINC codes for SARS-CoV-2 molecular tests were sourced from the Sentinel Initiative. SARS-CoV-2 antigen tests have been shown to have lower sensitivities and specificities than molecular tests, and were not considered. April 1, 2020 served as the study start date, as that is the date when the ICD code for COVID-19 (U07.1) was released by the CDC; before this, testing was also not widespread.

The baseline period was defined as the 365 days prior to the index date; individuals were only excluded if they have fewer than two encounters in EHR data AND fewer than two claims in insurance claims data within the baseline period (inclusive of the index date). Pediatric patients (under 18 years of age at their index date) were excluded, as the effects of COVID-19 are less pronounced in this population. Patients with no claims information after the index date were excluded to avoid mistakenly presuming patients were healthy after the index date because they have no claims data, when in reality they had experienced outcomes that were not recorded in the dataset (a form of misclassification bias).

Evidence of ICU and/or mechanical ventilation were identified via CPT/HCPCS codes as specified by the Sentinel Initiative.

Propensity score stratification was chosen as the method to balance cohorts in order to efficiently retain individuals in the analysis when handling imbalanced cohort sizes.

Several other propensity score balancing methods were considered, including 1:1 patient matching, inverse probability of treatment weighting (IPTW) with weights stabilized based on cohort size, standardized mortality ratio weighting (SMRW) with weights truncated at 8, and propensity score stratification using 500 bins.

For the individuals with COVID-19, stratified propensity weights ($w_{i}$) were calculated using the following formula:

$$w_{i}=\frac{n_{b}n_{COVID-19}}{n_{b,}{}_{COVID-19} N}$$

where $n_{b}$is the number of patients in bin *b*, $n_{COVID-19}$is the number of patients with COVID-19 in bin *b*, $n_{b,}{}_{COVID-19}$is the number of patients in bin *b* with COVID-19, and *N* is the total number of patients.

For individuals with influenza, stratified propensity weights ($w_{i}$) were calculated using the following formula:

$$w_{i}=\frac{n_{b}n_{influenza}}{n_{b,}{}_{influenza} N}$$

where $n_{influenza}$is the number of patients with influenza in bin *b*, and $n_{b,}{}_{influenza}$is the number of patients in bin *b* with influenza.

For each sensitivity analysis, propensity scores were recalculated (i.e. a new regression classifier was trained on the sub-cohort included in the sensitivity analysis), and new stratified weights were computed.

To assess the impact of unmeasured confounders, an E-value was calculated for the 8 primary and secondary endpoints. This E-value determines the minimum strength an unmeasured variable would have to be associated with both the exposure (influenza vs. COVID-19) and the outcome in order for there to be no difference in the two cohorts (or, for the value of 1.0 to fall within the 95% confidence interval of the calculated hazard ratio). These results are presented in Supplemental Table 4. Analysis was performed in R version 4.1.1, using the “EValue” package [[3]](https://paperpile.com/c/h4aej8/NkFHI).

# References

1. [Place of Service Code Set. [cited 20 Aug 2021]. Available:](http://paperpile.com/b/h4aej8/2OKz) <https://www.cms.gov/Medicare/Coding/place-of-service-codes/Place_of_Service_Code_Set>

2. [Dentali F, Di Micco G, Giorgi Pierfranceschi M, Gussoni G, Barillari G, Amitrano M, et al. Rate and duration of hospitalization for deep vein thrombosis and pulmonary embolism in real-world clinical practice. Ann Med. 2015;47: 546–554.](http://paperpile.com/b/h4aej8/TzRQw)

3. [Mathur MB, Ding P, Riddell CA, VanderWeele TJ. Web Site and R Package for Computing E-values. Epidemiology. 2018;29: e45–e47.](http://paperpile.com/b/h4aej8/NkFHI)
