# Supplementary material for: COVID-19 is associated with higher risk of venous thrombosis, but not arterial thrombosis, compared with influenza: Insights from a large US cohort
Source: PLoS One. 2022 Jan 12;17(1):e0261786. doi: 10.1371/journal.pone.0261786 (PMC8754296; doi:10.1371/journal.pone.0261786)
Supplement: S1 Table — (DOCX) [file pone.0261786.s002.docx]

Supplemental Table 1: ICD codes to define COVID-19 and influenza

​​

| **Diagnosis** | **ICD-10-CM** | **Description** |
| --- | --- | --- |
| COVID-19 | B9729 | Other coronavirus as the cause of diseases classified elsewhere |
| COVID-19 | U071 | COVID-19, virus identified [code effective April 1, 2020] |
| COVID-19 | B342 | Coronavirus infection, unspecified site |
| COVID-19 | B9721 | SARS-associated coronavirus as the cause of diseases classified elsewhere |
| COVID-19 | J1281 | Pneumonia due to SARS-associated coronavirus |
| Influenza | J09 | Influenza due to certain identified influenza viruses |
| Influenza | J09X | Influenza due to identified novel influenza A virus |
| Influenza | J09X1 | Influenza due to identified novel influenza A virus with pneumonia |
| Influenza | J09X2 | Influenza due to identified novel influenza A virus with other respiratory manifestations |
| Influenza | J09X3 | Influenza due to identified novel influenza A virus with gastrointestinal manifestations |
| Influenza | J09X9 | Influenza due to identified novel influenza A virus with other manifestations |
| Influenza | J10 | Influenza due to other identified influenza virus |
| Influenza | J100 | Influenza due to other identified influenza virus with pneumonia |
| Influenza | J1000 | Influenza due to other identified influenza virus with unspecified type of pneumonia |
| Influenza | J1001 | Influenza due to other identified influenza virus with the same other identified influenza virus pneumonia |
| Influenza | J1008 | Influenza due to other identified influenza virus with other specified pneumonia |
| Influenza | J101 | Influenza due to other identified influenza virus with other respiratory manifestations |
| Influenza | J102 | Influenza due to other identified influenza virus with gastrointestinal manifestations |
| Influenza | J108 | Influenza due to other identified influenza virus with other manifestations |
| Influenza | J1081 | Influenza due to other identified influenza virus with encephalopathy |
| Influenza | J1082 | Influenza due to other identified influenza virus with myocarditis |
| Influenza | J1083 | Influenza due to other identified influenza virus with otitis media |
| Influenza | J1089 | Influenza due to other identified influenza virus with other manifestations |
| Influenza | J11 | Influenza due to unidentified influenza virus |
| Influenza | J110 | Influenza due to unidentified influenza virus with pneumonia |
| Influenza | J1100 | Influenza due to unidentified influenza virus with unspecified type of pneumonia |
| Influenza | J1108 | Influenza due to unidentified influenza virus with specified pneumonia |
| Influenza | J111 | Influenza due to unidentified influenza virus with other respiratory manifestations |
| Influenza | J112 | Influenza due to unidentified influenza virus with gastrointestinal manifestations |
| Influenza | J118 | Influenza due to unidentified influenza virus with other manifestations |
| Influenza | J1181 | Influenza due to unidentified influenza virus with encephalopathy |
| Influenza | J1182 | Influenza due to unidentified influenza virus with myocarditis |
| Influenza | J1183 | Influenza due to unidentified influenza virus with otitis media |
| Influenza | J1189 | Influenza due to unidentified influenza virus with other manifestations |
