# Supplementary material for: COVID-19 is associated with higher risk of venous thrombosis, but not arterial thrombosis, compared with influenza: Insights from a large US cohort
Source: PLoS One. 2022 Jan 12;17(1):e0261786. doi: 10.1371/journal.pone.0261786 (PMC8754296; doi:10.1371/journal.pone.0261786)
Supplement: S2 Table — (DOCX) [file pone.0261786.s003.docx]

Supplemental Table 2: LOINC codes used to identify SARS-COV-2 and influenza molecular tests

| **Diagnosis** | **LOINC** | **Description** |
| --- | --- | --- |
| COVID-19 | 94306-8 | SARS coronavirus 2 RNA panel - Unspecified specimen by NAA with probe detection |
| COVID-19 | 94307-6 | SARS coronavirus 2 N gene [Presence] in Unspecified specimen by Nucleic acid amplification using primer-probe set N1 |
| COVID-19 | 94308-4 | SARS coronavirus 2 N gene [Presence] in Unspecified specimen by Nucleic acid amplification using primer-probe set N2 |
| COVID-19 | 94309-2 | SARS coronavirus 2 RNA [Presence] in Unspecified specimen by NAA with probe detection |
| COVID-19 | 94310-0 | SARS-like coronavirus N gene [Presence] in Unspecified specimen by NAA with probe detection |
| COVID-19 | 94311-8 | SARS coronavirus 2 N gene [Cycle Threshold #] in Unspecified specimen by Nucleic acid amplification using primer-probe set N1 |
| COVID-19 | 94312-6 | SARS coronavirus 2 N gene [Cycle Threshold #] in Unspecified specimen by Nucleic acid amplification using primer-probe set N2 |
| COVID-19 | 94313-4 | SARS-like coronavirus N gene [Cycle Threshold #] in Unspecified specimen by NAA with probe detection |
| COVID-19 | 94314-2 | SARS coronavirus 2 RdRp gene [Presence] in Unspecified specimen by NAA with probe detection |
| COVID-19 | 94315-9 | SARS coronavirus 2 E gene [Presence] in Unspecified specimen by NAA with probe detection |
| COVID-19 | 94316-7 | SARS coronavirus 2 N gene [Presence] in Unspecified specimen by NAA with probe detection |
| COVID-19 | 94500-6 | SARS coronavirus 2 RNA [Presence] in Respiratory specimen by NAA with probe detection |
| COVID-19 | 94502-2 | SARS-related coronavirus RNA [Presence] in Respiratory specimen by NAA with probe detection |
| COVID-19 | 94509-7 | SARS coronavirus 2 E gene [Cycle Threshold #] in Unspecified specimen by NAA with probe detection |
| COVID-19 | 94510-5 | SARS coronavirus 2 N gene [Cycle Threshold #] in Unspecified specimen by NAA with probe detection |
| COVID-19 | 94511-3 | SARS coronavirus 2 ORF1ab region [Cycle Threshold #] in Unspecified specimen by NAA with probe detection |
| COVID-19 | 94531-1 | SARS coronavirus 2 RNA panel - Respiratory specimen by NAA with probe detection |
| COVID-19 | 94532-9 | SARS-related coronavirus+MERS coronavirus RNA [Presence] in Respiratory specimen by NAA with probe detection |
| COVID-19 | 94533-7 | SARS coronavirus 2 N gene [Presence] in Respiratory specimen by NAA with probe detection |
| COVID-19 | 94534-5 | SARS coronavirus 2 RdRp gene [Presence] in Respiratory specimen by NAA with probe detection |
| COVID-19 | 94559-2 | SARS coronavirus 2 ORF1ab region [Presence] in Respiratory specimen by NAA with probe detection |
| COVID-19 | 94565-9 | SARS coronavirus 2 RNA [Presence] in Nasopharynx by NAA with non-probe detection |
| COVID-19 | 94639-2 | SARS coronavirus 2 ORF1ab region [Presence] in Unspecified specimen by NAA with probe detection |
| COVID-19 | 94640-0 | SARS coronavirus 2 S gene [Presence] in Respiratory specimen by NAA with probe detection |
| COVID-19 | 94641-8 | SARS coronavirus 2 S gene [Presence] in Unspecified specimen by NAA with probe detection |
| COVID-19 | 94642-6 | SARS coronavirus 2 S gene [Cycle Threshold #] in Respiratory specimen by NAA with probe detection |
| COVID-19 | 94643-4 | SARS coronavirus 2 S gene [Cycle Threshold #] in Unspecified specimen by NAA with probe detection |
| COVID-19 | 94644-2 | SARS coronavirus 2 ORF1ab region [Cycle Threshold #] in Respiratory specimen by NAA with probe detection |
| COVID-19 | 94645-9 | SARS coronavirus 2 RdRp gene [Cycle Threshold #] in Unspecified specimen by NAA with probe detection |
| COVID-19 | 94646-7 | SARS coronavirus 2 RdRp gene [Cycle Threshold #] in Respiratory specimen by NAA with probe detection |
| COVID-19 | 94647-5 | SARS-related coronavirus RNA [Presence] in Unspecified specimen by NAA with probe detection |
| COVID-19 | 94660-8 | SARS coronavirus 2 RNA [Presence] in Serum or Plasma by NAA with probe detection |
| COVID-19 | 94745-7 | SARS coronavirus 2 RNA |
| COVID-19 | 94746-5 | SARS coronavirus 2 RNA |
| COVID-19 | 94756-4 | SARS coronavirus 2 N gene |
| COVID-19 | 94757-2 | SARS coronavirus 2 N gene |
| COVID-19 | 94758-0 | SARS-related coronavirus E gene |
| COVID-19 | 94759-8 | SARS coronavirus 2 RNA |
| COVID-19 | 94760-6 | SARS coronavirus 2 N gene |
| COVID-19 | 94765-5 | SARS-related coronavirus E gene |
| COVID-19 | 94766-3 | SARS coronavirus 2 N gene |
| COVID-19 | 94767-1 | SARS coronavirus 2 S gene |
| COVID-19 | 94819-0 | SARS coronavirus 2 RNA |
| COVID-19 | 94845-5 | SARS coronavirus 2 RNA |
| COVID-19 | 95380-2 | Influenza virus A + B and SARS-CoV-2 (COVID-19) and SARS-related CoV RNA panel - Respiratory specimen by NAA with probe detection |
| COVID-19 | 95406-5 | SARS coronavirus 2 RNA |
| COVID-19 | 95409-9 | SARS coronavirus 2 N gene |
| COVID-19 | 95422-2 | Influenza virus A + B RNA and SARS-CoV-2 (COVID-19) N gene panel - Respiratory specimen by NAA with probe detection |
| COVID-19 | 95423-0 | Influenza virus A + B and SARS-CoV-2 (COVID-19) identified in Respiratory specimen by NAA with probe detection |
| COVID-19 | 95425-5 | SARS-CoV-2 (COVID-19) N gene [Presence] in Saliva (oral fluid) by NAA with probe detection |
| COVID-19 | 95521-1 | SARS coronavirus 2 N gene |
| COVID-19 | 95522-9 | SARS coronavirus 2 N gene |
| COVID-19 | 95608-6 | SARS coronavirus 2 RNA |
| Influenza | 344879 | Influenza virus A RNA [Presence] in Unspecified specimen by NAA with probe detection |
| Influenza | 409821 | Influenza virus B RNA [Presence] in Unspecified specimen by NAA with probe detection |
| Influenza | 485094 | Influenza virus A and B RNA [Identifier] in Unspecified specimen by NAA with probe detection |
| Influenza | 502195 | Respiratory pathogens DNA and RNA 12a panel - Unspecified specimen by NAA with probe detection |
| Influenza | 299073 | Haemophilus influenzae B DNA [Presence] in Unspecified specimen by NAA with probe detection |
| Influenza | 299099 | Parainfluenza virus 2 RNA [Presence] in Unspecified specimen by NAA with probe detection |
| Influenza | 299107 | Parainfluenza virus 3 RNA [Presence] in Unspecified specimen by NAA with probe detection |
| Influenza | 299065 | Haemophilus influenzae A DNA [Presence] in Unspecified specimen by NAA with probe detection |
| Influenza | 299081 | Parainfluenza virus 1 RNA [Presence] in Unspecified specimen by NAA with probe detection |
| Influenza | 410100 | Parainfluenza virus 4 RNA [Presence] in Unspecified specimen by NAA with probe detection |
| Influenza | 624627 | Influenza virus A+B RNA [Presence] in Unspecified specimen by NAA with probe detection |
| Influenza | 821710 | Parainfluenza virus 1 RNA [Presence] in Nasopharynx by NAA with non-probe detection |
| Influenza | 821660 | Influenza virus A RNA [Presence] in Nasopharynx by NAA with non-probe detection |
| Influenza | 821728 | Parainfluenza virus 2 RNA [Presence] in Nasopharynx by NAA with non-probe detection |
| Influenza | 821744 | Parainfluenza virus 4 RNA [Presence] in Nasopharynx by NAA with non-probe detection |
| Influenza | 821702 | Influenza virus B RNA [Presence] in Nasopharynx by NAA with non-probe detection |
| Influenza | 821736 | Parainfluenza virus 3 RNA [Presence] in Nasopharynx by NAA with non-probe detection |
| Influenza | 821678 | Influenza virus A H1 RNA [Presence] in Nasopharynx by NAA with non-probe detection |
| Influenza | 495242 | Influenza virus A H3 RNA [Presence] in Unspecified specimen by NAA with probe detection |
| Influenza | 495218 | Influenza virus A H1 RNA [Presence] in Unspecified specimen by NAA with probe detection |
| Influenza | 821694 | Influenza virus A H3 RNA [Presence] in Nasopharynx by NAA with non-probe detection |
| Influenza | 821686 | Influenza virus A H1 2009 pandemic RNA [Presence] in Nasopharynx by NAA with non-probe detection |
| Influenza | 554659 | Influenza virus A H1 2009 pandemic RNA [Presence] in Unspecified specimen by NAA with probe detection |
| Influenza | 554634 | Influenza virus A swine origin RNA [Identifier] in Unspecified specimen by NAA with probe detection |
| Influenza | 760785 | Influenza virus A RNA [Presence] in Nasopharynx by NAA with probe detection |
| Influenza | 760801 | Influenza virus B RNA [Presence] in Nasopharynx by NAA with probe detection |
| Influenza | 770289 | Influenza virus A H1 2009 pandemic RNA [Presence] in Nasopharynx by NAA with probe detection |
| Influenza | 805903 | Influenza virus A H3 HA gene [Presence] in Nasopharynx by NAA with probe detection |
| Influenza | 770263 | Influenza virus A H1 RNA [Presence] in Nasopharynx by NAA with probe detection |
| Influenza | 770271 | Influenza virus A H3 RNA [Presence] in Nasopharynx by NAA with probe detection |
| Influenza | 495317 | Influenza virus A RNA [Presence] in Isolate by NAA with probe detection |
| Influenza | 613661 | Haemophilus influenzae DNA [Presence] in Unspecified specimen by NAA with probe detection |
| Influenza | 495358 | Influenza virus B RNA [Presence] in Isolate by NAA with probe detection |
