# Supplementary material for: COVID-19 is associated with higher risk of venous thrombosis, but not arterial thrombosis, compared with influenza: Insights from a large US cohort
Source: PLoS One. 2022 Jan 12;17(1):e0261786. doi: 10.1371/journal.pone.0261786 (PMC8754296; doi:10.1371/journal.pone.0261786)
Supplement: S3 Table — Standardized differences for sensitivity cohorts are available on request. (DOCX) [file pone.0261786.s004.docx]

Supplemental Table 3: Standardized differences of cohort characteristics before and after weighting, for the primary (full) cohort. Standardized differences for sensitivity cohorts are available on request.

|  | | COVID-19 cohort | **Influenza**  **cohort** |  |  |
| --- | --- | --- | --- | --- | --- |
|  |  | N = 417,969  (after trimming the tails) | N = 345,934  (after trimming the tails) | *Standardized difference (before weighting)* | *Standardized difference (after weighting)* |
| Age | 18-44 | 128,424 (31) | 161,165 (47) | 0.33 | 0.011 |
|  | 45-54 | 60,179 (14) | 58,547 (17) | 0.07 | 0.006 |
|  | 55-64 | 73,553 (18) | 62,051 (18) | 0.01 | 0.010 |
|  | 65-74 | 64,416 (15) | 34,519 (10) | 0.16 | 0.010 |
|  | 75-84 | 50,892 (12) | 18,382 (5.3) | 0.24 | 0.000 |
|  | ≥85 | 40,505 (9.7) | 11,270 (3.3) | 0.26 | 0.015 |
| Sex | Male | 160,795 (38) | 118,043 (34) | 0.09 | 0.007 |
|  | Female | 256,710 (61) | 227,594 (66) | 0.09 | 0.007 |
|  | Other/unknown | 464 (0.11) | 297 (0.086) | 0.01 | 0.008 |
| Severity of infection  ***Time frame:*** *date of diagnosis (start of hospitalization) until end of hospitalization* | Not hospitalized | 371,878 (89) | 328,280 (95) | 0.22 | 0.079 |
|  | Hospitalized, no evidence of ICU/ventilator during hospitalization | 33,437 (8) | 12,863 (3.7) | 0.18 | 0.065 |
|  | Hospitalized with evidence of ICU/ventilator during hospitalization | 12,654 (3) | 4,791 (1.4) | 0.11 | 0.041 |
| Care setting of diagnosis  ***Time frame:*** *date of diagnosis* | Ambulatory/outpatient | 194,346 (46) | 252,001 (73) | 0.56 | 0.359 |
|  | Hospital | 46,091 (11) | 17,654 (5.1) | 0.22 | 0.079 |
|  | ED | 49,611 (12) | 56,025 (16) | 0.12 | 0.103 |
|  | SNF or long-term care | 15,244 (3.6) | 1,206 (0.35) | 0.24 | 0.120 |
|  | Unknown/not reported | 112,677 (27) | 19,048 (5.5) | 0.61 | 0.483 |
| Recent institutional stay encounter (90 - 1 day before index) | Yes | 53,607 (13) | 18,001 (5.2) | 0.27 | 0.003 |
| ***Time frame:*** *90 - 1 day before diagnosis* | No | 364,362 (87) | 327,933 (95) | 0.27 | 0.003 |
| Baseline medications/transfusions  ***Time frame:*** *183 - 3 days before diagnosis* | Anticoagulants | 10,079 (2.4) | 6,401 (1.9) | 0.04 | 0.007 |
|  | Antiplatelet | 18,887 (4.5) | 11,397 (3.3) | 0.06 | 0.012 |
|  | Statins | 64,733 (15) | 45,419 (13) | 0.07 | 0.010 |
|  | Oral chemotherapeutics | 5,312 (1.3) | 11,169 (3.2) | 0.13 | 0.007 |
|  | Tamoxifen | 2,179 (0.52) | 2,166 (0.63) | 0.01 | 0.002 |
|  | Oral contraceptives | 4,979 (1.2) | 12,088 (3.5) | 0.15 | 0.010 |
|  | Estrogen replacement | 58 (0.014) | 146 (0.042) | 0.02 | 0.002 |
|  | Testosterone replacement | 522 (0.12) | 1,137 (0.33) | 0.04 | 0.003 |
| Baseline comorbidities  ***Time frame:***  *365 - 1 day before diagnosis for comorbidities,*  *7 days before - 7 days after diagnosis for labs* | Cardiovascular disease | 225,980 (54) | 142,789 (41) | 0.26 | 0.001 |
|  | Venous thromboembolism | 14,056 (3.4) | 6,614 (1.9) | 0.09 | 0.008 |
|  | Neurologic disease that promotes stasis/immobility | 57,662 (14) | 15,253 (4.4) | 0.33 | 0.001 |
|  | Obesity | 104,144 (25) | 84,457 (24) | 0.01 | 0.011 |
|  | Alcohol abuse | 11,816 (2.8) | 7,767 (2.2) | 0.04 | 0.000 |
|  | Current tobacco use | 56,925 (14) | 59,818 (17) | 0.10 | 0.009 |
|  | Pregnancy | 14,435 (3.5) | 22,349 (6.5) | 0.14 | 0.008 |
|  | Chronic kidney disease | 66,574 (16) | 29,906 (8.6) | 0.22 | 0.016 |
|  | cancer | 35,379 (8.5) | 26,486 (7.7) | 0.03 | 0.005 |
|  | COPD | 54,389 (13) | 40,825 (12) | 0.04 | 0.007 |
|  | diabetes | 110,485 (26) | 56,633 (16) | 0.25 | 0.010 |
|  | hyperlipidemia | 154,459 (37) | 100,661 (29) | 0.17 | 0.009 |
|  | hypertension | 195,897 (47) | 119,970 (35) | 0.25 | 0.004 |
|  | rheumatic disease | 18,009 (4.3) | 15,997 (4.6) | 0.02 | 0.006 |
|  | atrial fibrillation | 32,360 (7.7) | 14,331 (4.1) | 0.15 | 0.010 |
|  | antiphospholipid antibody syndrome | 356 (0.085) | 334 (0.097) | 0.00 | 0.000 |
|  | inherited thrombophilia | 904 (0.22) | 755 (0.22) | 0.00 | 0.003 |
|  | ischemic stroke | 17,827 (4.3) | 5,560 (1.6) | 0.16 | 0.011 |
|  | myocardial infarction | 8,042 (1.9) | 3,639 (1.1) | 0.07 | 0.009 |
|  | heart failure | 44,876 (11) | 17,687 (5.1) | 0.21 | 0.021 |
|  | peripheral arterial disease | 31,758 (7.6) | 9,418 (2.7) | 0.22 | 0.010 |
|  | Polycythemia (via ICD or hemoglobin >16 g/dL) | 1,483 (0.35) | 1,273 (0.37) | 0.00 | 0.001 |
|  | Thrombocytosis (via ICD or platelet count >450 x 10^9/L) | 1,973 (0.47) | 1,378 (0.4) | 0.01 | 0.000 |
