# Supplementary material for: COVID-19 is associated with higher risk of venous thrombosis, but not arterial thrombosis, compared with influenza: Insights from a large US cohort
Source: PLoS One. 2022 Jan 12;17(1):e0261786. doi: 10.1371/journal.pone.0261786 (PMC8754296; doi:10.1371/journal.pone.0261786)
Supplement: S4 Table — (DOCX) [file pone.0261786.s005.docx]

Supplemental Table 4: Unadjusted event rates for components of secondary composite endpoints

|  | | COVID-19 cohort | | **Influenza cohort** | |
| --- | --- | --- | --- | --- | --- |
|  |  | Absolute risk  (N, %) | Incidence rates (per person-year) | Absolute risk (N, %) | Incidence rates (per person-year) |
|  | Angina | 1,692 (0.4) | 0.03 | 1,353 (0.39) | 0.02 |
|  | Peripheral arterial disease | 2,917 (0.7) | 0.04 | 1,306 (0.38) | 0.02 |
|  | Amputation | 1,321 (0.32) | 0.02 | 550 (0.16) | 0.01 |
|  | Coronary angioplasty | 2,760 (0.66) | 0.04 | 2,517 (0.73) | 0.03 |
|  | Coronary artery bypass grafting | 4,690 (1.1) | 0.07 | 3,428 (0.99) | 0.04 |
|  | Venous thrombosis of devices, implants, or grafts | 309 (0.074) | 0.00 | 153 (0.044) | 0.00 |
