# Supplementary material for: COVID-19 is associated with higher risk of venous thrombosis, but not arterial thrombosis, compared with influenza: Insights from a large US cohort
Source: PLoS One. 2022 Jan 12;17(1):e0261786. doi: 10.1371/journal.pone.0261786 (PMC8754296; doi:10.1371/journal.pone.0261786)
Supplement: S5 Table — NOTE: for endpoints for which hazard ratios were not statistically significant, no E-values were calculated. (DOCX) [file pone.0261786.s006.docx]

Supplemental Table 5: E-values (point estimate and 95% confidence intervals) for primary and secondary outcomes. NOTE: for endpoints for which hazard ratios were not statistically significant, no E-values were calculated.

|  |  |
| --- | --- |
|  | E value, point estimate (95% CI) |
| Primary arterial endpoint | NA |
| Primary venous endpoint | 2.43 (2.1) |
| Ischemic stroke | NA |
| Myocardial infarction | NA |
| Deep vein thrombosis | 2.06 (1.67) |
| Pulmonary embolism | 3.04 (2.52) |
| Secondary arterial endpoint | 1.53 (1.39) |
| Secondary venous endpoint | 2.39 (2.06) |
