# Supplementary material for: COVID-19 is associated with higher risk of venous thrombosis, but not arterial thrombosis, compared with influenza: Insights from a large US cohort
Source: PLoS One. 2022 Jan 12;17(1):e0261786. doi: 10.1371/journal.pone.0261786 (PMC8754296; doi:10.1371/journal.pone.0261786)

Supplemental Figure 1: standardized difference, visualized, for different propensity score methods (before and after balancing).

Abbreviations: IPTW - inverse probability of treatment weighting; SMRW - standardized mortality ratio weighting


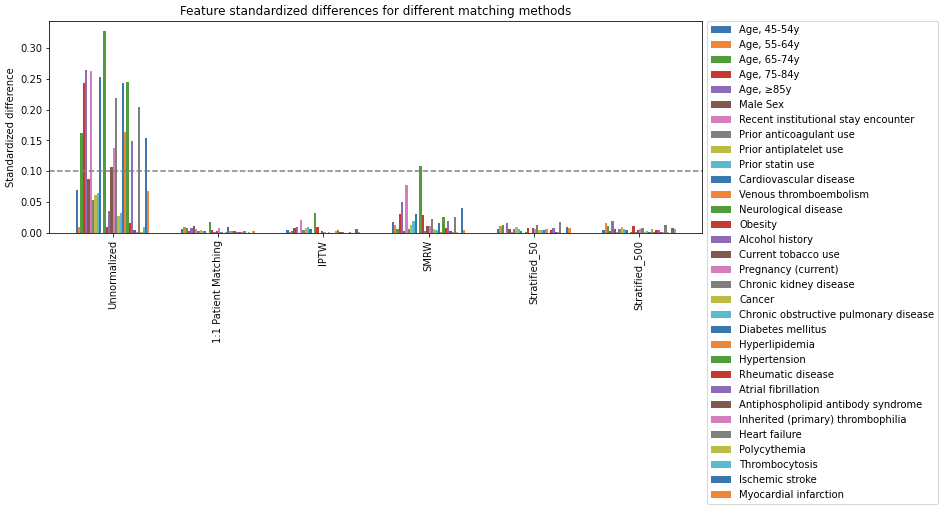

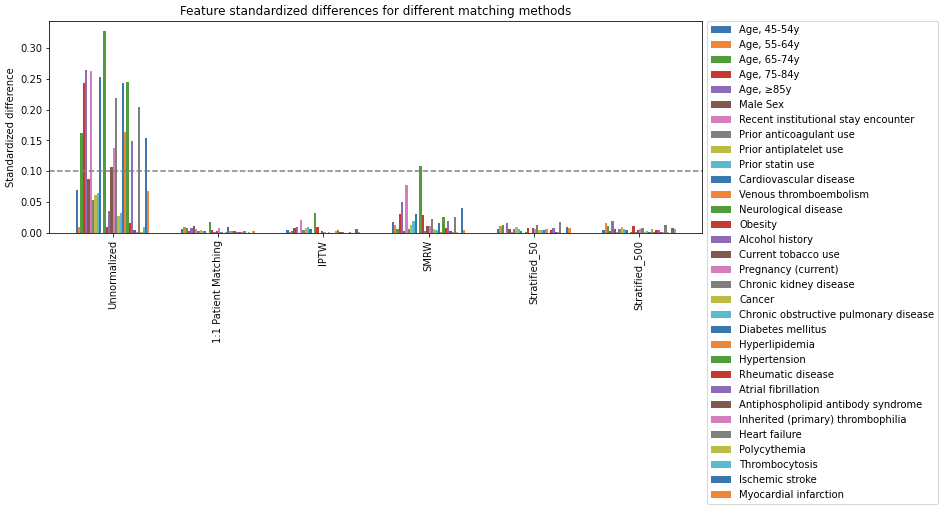

Supplement: S1 Fig — Abbreviations: IPTW—inverse probability of treatment weighting; SMRW—standardized mortality ratio weighting. (DOCX) [file pone.0261786.s007.docx]
